# Pro- and anti-apoptotic CD95 signaling in T cells

**DOI:** 10.1186/1478-811X-9-7

**Published:** 2011-04-08

**Authors:** Maren Paulsen, Ottmar Janssen

**Affiliations:** 1 Christian-Albrechts-University of Kiel Institute of Immunology, University Hospital Schleswig-Holstein Campus Kiel, Arnold-Heller-Str. 3 Bldg 17, D-24105 Kiel, Germany

## Abstract

The TNF receptor superfamily member CD95 (Fas, APO-1, TNFRSF6) is known as the prototypic death receptor in and outside the immune system. In fact, many mechanisms involved in apoptotic signaling cascades were solved by addressing consequences and pathways initiated by CD95 ligation in activated T cells or other "CD95-sensitive" cell populations. As an example, the binding of the inducible CD95 ligand (CD95L) to CD95 on activated T lymphocytes results in apoptotic cell death. This activation-induced cell death was implicated in the control of immune cell homeostasis and immune response termination. Over the past years, however, it became evident that CD95 acts as a dual function receptor that also exerts anti-apoptotic effects depending on the cellular context. Early observations of a potential non-apoptotic role of CD95 in the growth control of resting T cells were recently reconsidered and revealed quite unexpected findings regarding the costimulatory capacity of CD95 for primary T cell activation. It turned out that CD95 engagement modulates TCR/CD3-driven signal initiation in a dose-dependent manner. High doses of immobilized CD95 agonists or cellular CD95L almost completely silence T cells by blocking early TCR-induced signaling events. In contrast, under otherwise unchanged conditions, lower amounts of the same agonists dramatically augment TCR/CD3-driven activation and proliferation. In the present overview, we summarize these recent findings with a focus on the costimulatory capacity of CD95 in primary T cells and discuss potential implications for the T cell compartment and the interplay between T cells and CD95L-expressing cells including antigen-presenting cells.

## Introduction

Members of the 'tumour necrosis factor receptor' (TNFR) superfamily and their ligands are crucial regulators of cellular activation and death. According to their structural composition and/or cellular function, the TNFR family can be further divided into the three subgroups of 'death domain' (DD)-containing receptors, 'TNFR-associated factor' (TRAF) binding receptors and decoy receptors. The eponymous 'TNF receptor-1' (TNFR-1, TNFRSF1), CD95 (Fas, APO-1, TNFRSF6) and 'TNF-related apoptosis inducing ligand' (TRAIL) receptors (DR4/TNFRSF10A, DR5/TNFRSF10B), contain cytoplasmic death domains, which are essential for the direct induction of cell death. In contrast, the TNFR family members TNFR-2, CD27, 4-1BB (CD137), OX-40 (CD134), 'herpesvirus entry mediator' (HVEM), CD30 and 'glucocorticoid-induced TNFR family related protein' (GITR) belong to the subgroup of TRAF binding receptors that lack a characteristic DD, but harbor 4-6 amino acids important for the recruitment of TRAF proteins. These receptors have been mainly implicated in non-apoptotic processes including cellular activation, differentiation and survival [1], but they might also be involved in other forms of cell death, e.g. programmed necrosis as in the case of TNFR-2 [2].

Although the DD-containing receptors have been mainly associated with the induction of apoptosis, these receptors can also exert non-apoptotic functions in a wide range of different cell populations. Thus, several "death receptors" have been implicated in the signal induction for activation, migration, proliferation or differentiation. As an example, agonistic anti-CD95 antibodies caused massive CD95-induced hepatitis in normal mice but increased liver regeneration in mice subjected to hepatectomy [3]. For TNFR-1, it was proposed that receptor internalization and the formation of TNF receptosomes transmit pro-apoptotic signals, whereas plasma membrane-associated receptors trigger non-apoptotic signaling to activate 'nuclear factor 'kappalight-chain-enhancer' of activated B-cells' (NF-κB) [4]. Thus, the very same DD receptors can exert pro- or anti-apoptotic effects in a context-specific fashion and maybe depending on receptor clustering and internalization or on signaling thresholds governed by other simultaneous cell-cell-interactions.

### CD95 - the prototype of a death receptor

The 45 kDa type-I transmembrane protein CD95 is a member of TNFR family and serves as the prototypic death receptor for the immune system. CD95-dependent apoptosis is triggered by CD95L (FasL, APO-1L, TNFSF6) binding and clustering of surface CD95. Oligomerization initiates the recruitment of the 'Fas (CD95) associated protein with death domain' (FADD) and procaspase-8 to form the 'death-inducing signaling complex' (DISC) [5,6]. In this multimolecular complex, procaspase-8 undergoes autocatalytic cleavage resulting in the generation of active caspase-8, which in turn regulates the extrinsic pathway leading to apoptotic cell death [7,8].

### CD95: a death receptor for lymphocyte homeostasis

The importance of the CD95/CD95L-system for lymphocyte homeostasis became apparent from the initial observation that naturally occurring mice which developed massive lymphadenopathy and suffered from lymphoproliferative syndromes carried the causative mutations in the genes encoding either for CD95 (*lpr *= lymphoproliferation) or for CD95L (*gld *= generalized lymphoproliferative disease). In both types of mice, the impaired CD95/CD95L-interaction resulted in an accumulation of unconventional T cells (Thy-1^+^CD4^-^CD8^-^TCRα/β^+^B220^+^) as well as in increased numbers of conventional B cells and CD4^+ ^and CD8^+ ^T cells [9-11]. This clearly suggested that signaling through the death receptor CD95 governs homeostasis of the lymphoid system. Since the observed pathology was apparently caused by a defective killing/dying capacity affecting both immature and mature cell populations, the phenotype might however have better been summarized as lymphoaccumulation rather than lymphoproliferation. Although functional defect mutants of CD95 or CD95L are rare in humans, the consequences of an impaired removal of pre-activated potentially dangerous cells are also characteristic for patients suffering from certain types of autoimmune lymphoproliferative syndrome (ALPS) [12,13].

In essence, *lpr/gld*- or ALPS-pathologies were associated with an impaired 'activation-induced cell death' (AICD). Briefly, AICD describes an activation-driven death that is associated with an induced increase in CD95L expression (e.g. following multiple TCR stimulations) which in turn results in suicidal or fratricidal CD95-mediated apoptosis. There is no doubt that death receptors play a prominent role in the development of AICD sensitivity [14,15]. However, it was also reported that death receptor independent signals via the TCR contribute to apoptosis sensitivity. One mechanism involves the cleavage of the 'hematopoietic progenitor kinase-1' (HPK-1), which in turn binds to the 'inhibitor of κB (IκB) kinase' (IKK) complex and thereby interferes with pro-survival signaling by NF-κB [16]. Notably, activated T cells which are not restimulated die by 'activated cell autonomous death' (ACAD), a process which is also termed passive cell death or death by neglect and can also be observed under conditions of cytokine withdrawal [17].

Both ACAD and AICD have been implicated in thymocyte selection and the termination of the immune responses providing an effective means for the removal of useless cells. If one of the control mechanisms is missing, due to functional impairment of the trigger system or the involved signaling molecules, immature or mature cells might escape immune selection, and potentially dangerous autoreactive cells accumulate in lymphoid organs and in the periphery. Therefore, mutations in several genes encoding for apoptosis regulators (e.g. ligands, receptors, adapter proteins or caspases) cause immune dysfunction and severe autoimmunity as observed in ALPS patients and respective animal models.

Death receptors such as CD95 trigger the extrinsic apoptosis pathway. They deliver pro-apoptotic signals at the plasma membrane by formation of a DISC and by subsequent direct activation of a proteolytic caspase signaling cascade in so-called 'type I' cells or via an additional (intrinsic) mitochondrial amplification loop in 'type II' cells. As mentioned, the earlier studies on AICD suggested a prominent role of CD95 in this context [6,14]. However, more recent studies propose that the pro-apoptotic BH3-only protein 'Bcl-2-interacting mediator of cell death' (Bim), a member of the Bcl-2 protein family and thus a mediator of the mitochondrial apoptosis route, is also involved in the deletion of peripheral T cells [18-21]. Therefore, it has to be concluded that CD95 and Bim play a synergistic or cooperative role in the contraction phase of T cell responses, and link the different branches of the intracellular apoptosis machinery [22].

### CD95 - a dual-function receptor

Apart from its primary pro-apoptotic role, follow-up studies revealed that CD95 might rather act as a dual-function signaling receptor with tissue-specific functions and give rise to pro- and anti-apoptotic signals depending on the cellular microenvironment [8]. Thus, CD95 was found to affect proliferation, differentiation and migration processes as well as cytokine production in different hematopoietic and non-hematopoietic cell types. Moreover, a very recent study discovered that membrane-bound CD95L is essential for triggering cytotoxic activity, whereas soluble CD95L (generated when mCD95L is proteolytically cleaved by matrix metalloproteinases [23]) primarily promotes non-apoptotic activities [24] and even neutrophil chemotaxis [25].

As an apostil, non-apoptotic regulatory functions of the CD95L have also been suggested. This phenomenon, referred to as reverse or retrograde signaling, has been documented for several TNF ligands and increases the levels of complexity and plasticity during pro-and-anti-apoptotic cell-cell communication. Here, CD95 operates as the ligand for membrane-bound CD95L to induce signal alterations in the CD95L-expressing cell. However, the available data on the role of CD95L reverse signaling in T cells are still fragmentary. What is known is that reverse signaling in different T cell subsets requires a presumably simultaneous TCR/CD3-engagement. In terms of outcome and signaling pathways, the reported findings are, however, still somewhat confusing since they span from promotion of proliferation to cell cycle arrest [23,26-30].

As indicated, anti-apoptotic effects of CD95 seem to depend on several parameters including the cell type and cellular context, the mode of ligation, and the activation-associated signaling threshold. However, non-apoptotic or even costimulatory consequences of CD95 engagement were only sporadically reported for instance during liver regeneration, development and functional recovery of the central nervous system or neurite outgrowth, and the proliferation of growth factor deprived fibroblasts [31]. Only recently, it became evident that CD95 ligation might be highly relevant for the modulation of TCR/CD3 signaling in primary T cells.

### CD95 as a non-apoptotic costimulatory molecule for T cells

The textbooks tell that three signals are required for full activation and differentiation of resting T cells. The first signal emerges from an engagement of the TCR/CD3 complex, the second costimulatory signal is provided through the ligation of "classical" costimulatory receptors including CD28 or CD278 (ICOS) and the third signal for differentiation is provided by locally available cytokines. However, in recent years, TRAF binding receptors were identified as a second class of costimulatory receptors [1].

Based on experiments in *gld*- and *lpr*-mice, Alderson and colleagues provided first evidence for a potential role of CD95 in the activation of human T cells in 1993 when they reported a stronger proliferation and cytokine production in the presence of a functional CD95L/CD95-system [32]. It was subsequently reported that CD95-mediated costimulation involved activation of caspases in the absence of apoptosis [33,34]. However, since T cell activation through the TCR alone apparently also depended on caspase processing, it was concluded that caspase activation plays a more general role in proliferative processes and under such costimulatory conditions might not necessarily be associated with death receptor signaling [34-36]. Along this line, caspase-8 mutations in humans were found to be associated with severe T cell associated immune dysfunction [37]. Whereas cleavage of the classical caspase substrate 'Poly(ADP-Ribose) Polymerase' (PARP) in this scenario has been initially observed by Alam et al. [34], recent data from others and our own experiments rather point to a non-apoptotic caspase activation that spares cleavage of apoptosis-relevant substrates such as PARP [38]. More precisely, we demonstrated an incomplete cleavage of caspase-3 resulting in an accumulation of the p20 fragment under costimulatory conditions. Concomitantly, p17 and p19 fragments, which are characteristic for caspase-3 activity in apoptotic cells, were not seen in TCR/CD3-stimulated cells in the presence of CD95 agonists. We conclude that caspase activation is required for proliferation, but that non- or pro-apoptotic caspase activation is qualitatively different [35,38,39]. In view of our recent observation that the 'X-linked-inhibitor-of-apoptosis-protein' (XIAP) can interact with caspase-3 and thereby block full caspase activation [38], we suggest that in naïve T cells, CD95 initiates an incomplete cleavage of caspase-3, thereby presumably hindering the cleavage of pro-apoptotic substrates. Thus, caspase-3 activation in this scenario might affect different substrates, which in turn are crucial for supporting a proliferative response. However, different caspase substrate repertoires associated with proliferation and apoptosis, respectively, have to be proven and characterized. So far, only limited numbers of putative anti-apoptotic caspase substrates have been described that include RasGAP [40] or HPK-1 [41-43]. In this context, a very recent study describe a systematic computational screening method of caspase cleavage sites to provide more insight into the substrate specificity of caspases and facilitate the discovery of putative novel substrates [44].

One mechanism to balance caspase activity during survival and cell death has been discussed by Kurokawa and Kornbluth [45]. In their review, they summarize how phosphorylation can alter activities of both caspases and their potential targets (e.g. kinases and phosphatases), and how these classes of signaling molecules are linked to control apoptosis and survival. As an example, phosphorylation of nuclear caspase-2 at Ser122 by a DNA-dependent protein kinase induces non-apoptotic activation of the caspase and results in cell cycle arrest at a G2/M DNA damage checkpoint to allow DNA repair [46]. Thus, several caspases appear to be versatile enzymes with multiple functions beyond cell death induction depending on posttranslational modifications. Interestingly, in the context of non-apoptotic caspase activation by CD95 costimulation, a contribution to altered phosphorylation of caspases und their substrates has not been investigated. Furthermore, it still has to be clarified for non-apoptotic signaling, whether CD95 directly recruits or activates caspases via its adapter molecule FADD or whether CD95 only supports the TCR-mediated activation of caspases via a signaling complex formed for example by 'B-cell CLL/lymphoma associated 10' (Bcl-10), 'CARD-containing MAGUK protein1' (CARMA1) and 'mucosa-associated lymphoid tissue-1' (MALT-1) [47].

An additional level of regulation of life and death decisions in T cells is represented by the molecule 'cellular FLICE (caspase 8)-like inhibitory protein' (cFLIP). cFLIP can inhibit death receptor signaling and caspase-8 activation, possibly through competition with caspase-8 for the recruitment to FADD. However, it has also been shown that cFLIP mediates activation of full-length caspase-8 at the DISC, which in turn results in caspase-8-induced cleavage of cFLIP. Apparently, this process requires the cleavage of cFLIP to a p43-fragment [48]. More recently, it was argued that p22-cFLIP (but not p43) can activate NF-κB by directly interacting with the IKK complex [49]. Cleaved cFLIP then allows a more efficient recruitment of TRAF1/2, the 'receptor-interacting protein' (RIP1) and the the 'rapidly growing fibrosarcoma or rat fibrosarcoma-1' (Raf-1) protein to the cFLIP-caspase-8 heterodimer. This complex supports the subsequent activation of NF-κB and 'mitogen activated protein kinase' (MAPK) [50], both acting as key mediators of inflammatory or proliferative responses and survival. Since also Bcl-10 and MALT-1 associate to this complex [51], cFLIP could be considered as the candidate for determining life and death by directly linking CD95-costimulatory signals to TCR signaling pathways.

So far, three cFLIP isoforms (cFLIP_L_, cFLIP_S _and cFLIP_R_) were identified, with cFLIP_S/R _presumably mediating a block in apoptosis by inhibiting caspase-8 at the DISC as mentioned above. The role of cFLIP_L _regarding an inhibition at the DISC is still a matter of debate and seems to be regulated at the level of expression [49,51,52]. Likewise, the role of cFLIP in the activation of NF-κB might be much more complex than described above. In fact, several groups reported inhibitory effects of cFLIP on CD95-induced NF-κB activation [53-55]. Further downstream of the CD95 apoptotic pathway, anti-apoptotic proteins including Bcl-2/Bcl-X_L _and XIAP may prevent apoptosis [7,38]. An increased expression of anti-apoptotic checkpoint proteins in CD95-sensitive cells could therefore shift caspase-associated death signaling to NF-κB-associated proliferative signaling pathways in the course of cellular activation [56,57].

### The two faces of non-apoptotic CD95 signaling in T cells

As stated above, several earlier data pointed to a potential modulation of T cell activation by CD95 [32,33]. However, the molecular mechanism of the CD95 costimulatory function had never been elucidated in detail. Moreover, the published data about a "CD95 costimulation" have been somehow inconsistent, since CD95 has been described as a silencer or an enhancer of primary human T cell activation [32,33,36,50,58,59].

**Figure 1:**
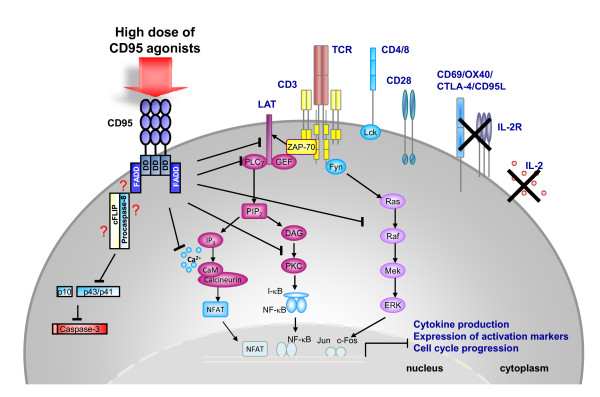
**CD95L-expressing APC down-modulate T cell responses**. High density CD95L, as expressed on transfectants or certain activated APC or mimicked by high amounts of CD95 agonists *in vitro*, engages membrane CD95 and interferes with proximal TCR signaling by inhibiting the phosphorylation of ZAP-70, PLCγ and PKC, leading to inefficient nuclear translocation of transcription factors like NFAT, NF-κB and AP-1 (Jun/c-Fos). CD95 engagement under such conditions also prevents activation of caspases and MAPK as well as Ca^2+^-mobilization. Subsequently, TCR-induced cytokine production and upregulation of activation markers are impaired, resulting in a CD95L-mediated complete block of cell cycle progression in naïve T cells.

It was proposed that the expression of CD95L on 'antigen-presenting cells' (APC) would be responsible for antigen-specific deletion of primed peripheral T cells, eventually leading to T cell tolerance or immunosuppression [60-62]. Follow-up studies, however, provided evidence for alternate activities of APC-associated CD95L on naïve T cells, including the complete block of T cell activation and proliferation in the absence of apoptosis [59,63]. Of note, it is not yet clear whether the block in caspase-8 activation observed under such conditions is due to an impaired recruitment to the DISC or whether caspase-8 activation is directly abrogated in the CD95 complex. This suppression of T cell activation affected several proximal TCR signaling pathways including the recruitment and phosphorylation of 'ζ-chain-associated protein of 70 kDa' (ZAP70), 'phospholipase Cγ' (PLCγ), and 'protein kinase C-Θ' (PKC-Θ) into lipid rafts, thereby preventing the formation of a functional signaling platform. Furthermore, CD95-costimulated T cells displayed inefficient nuclear translocation of transcription factors including the 'nuclear factor of activated T cells' (NFAT), NF-κB and the 'activator protein-1 (AP-1), reduced Ca^2+^-mobilization and decreased MAPK and caspase activation. As a consequence, Strauss and colleagues reported a down-regulation of activation markers and reduced secretion of several cytokines including IL-2, IFNγ or TNFα (Figure 1). This block in cell activation through CD95 is in line with an earlier report by Chen and co-workers, who described an inhibition of T cell proliferation by "CD95L-painted" K562/B7-1 cells that was only partially caused by apoptosis induction [58]. However in both studies, a positive costimulatory effect of CD95L had not been noted.

**Figure 2:**
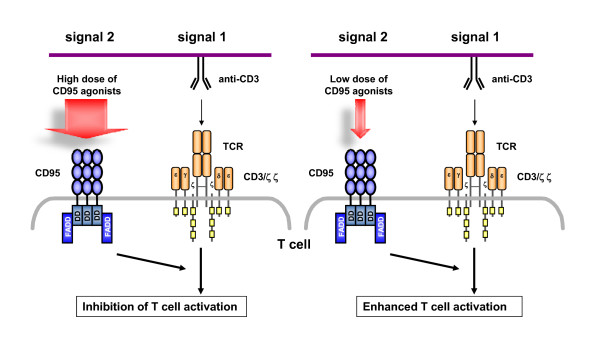
**Dose-dependent effects of CD95 coligation on primary T cell activation**. CD95 is capable of transducing non-apoptotic costimulatory signals in TCR/CD3-stimulated naïve T cells. Interestingly, the outcome of CD95 costimulation depends on the dose of agonist. Whereas high concentrations of CD95 agonists silence T cells, low doses augment TCR-induced activation and proliferation. Thus CD95 can act as a silencer or enhancer of primary T cell activation (see text for detail).

It should be emphasized that this "negative costimulation" is in full agreement with our own observations employing high concentrations of immobilized CD95 agonists and thus reflects the inhibitory branch of CD95 signaling. However, our titration experiments revealed that low doses of the very same CD95 agonists drastically augment T cell activation and proliferation under otherwise unchanged conditions. This indicates that the outcome of CD95 ligation on naïve T cells largely depends on the "dose of agonist", resulting in opposite effects from a complete block of activation (at high doses) to prominent costimulatory activation (at lower concentrations) (Figure 2). Surprisingly, low doses of CD95 agonists promote cell cycle progression in a much higher portion of a given T cell population compared to conventional costimulation through the classical costimulatory molecule CD28. At the level of surface appearance of activation markers and regarding the activation of cell cycle regulatory proteins, CD95 triggering might in fact replace the conventional "signal 2" [39].

**Figure 3:**
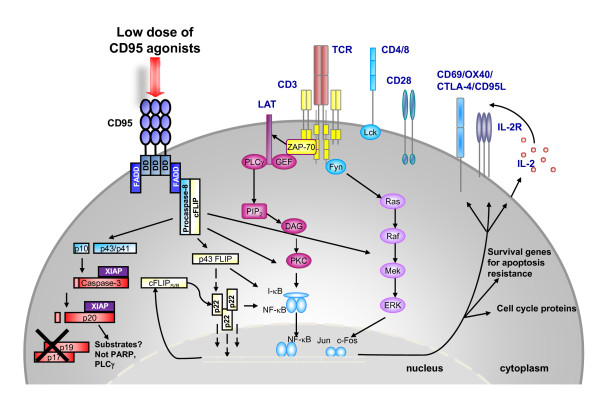
**Costimulation with low doses of CD95 agonists increases primary T cell activation**. CD95 coligation enhances MAPK and NF-κB activation in TCR-triggered cells and results in accelerated induction of activation markers, cell cycle regulatory proteins, cytokine secretion and cell cycle progression. The incomplete cleavage of caspase-3 into p20 fragments (possibly achieved by an interaction with XIAP) seems to be characteristic for non-apoptotic caspase activation and becomes more prominent upon CD95 costimulation. In line with the observed upregulation of anti-apoptotic proteins including cFLIP_R/S _and Bcl-X_L _in the presence of low dose CD95 agonists, CD95/TCR-stimulated cells display a partial apoptosis resistance.

In our recent report, we provided a detailed analysis of the signaling events associated with the positive costimulatory activity of CD95 [39] that complemented the inhibitory features of the CD95/CD95L-system described by Strauss and colleagues [59]. Thus, CD95 ligation at low agonist concentrations promotes TCR-triggered MAPK phosphorylation, non-apoptotic caspase and NF-κB activation and the upregulation of activation markers and anti-apoptotic checkpoint proteins [36,39,50]. Furthermore, our analyses indicate an accelerated actin-dependent CD95 and TCR co-internalization as a mechanism to establish receptor interference and signaling crosstalk [39]. As a consequence, enhanced cell cycle progression and proliferation are associated with an increased cytokine production (IFNγ, TNFα, IL-2) [32,33,36,39,50] (Figure 3).

Given that cFLIP and the Bcl-2 family member Bcl-X_L _(both upregulated in response to low concentrations of CD95 agonists [39]) are known for their capacity to interfere with apoptotic cell death [7,64,65], we observed a more generalized partial apoptosis resistance upon primary TCR/CD3 activation, which was further enhanced upon costimulation via CD95 [39]. Interestingly in this context, it has recently been shown that the anti-apoptotic cFLIP isoforms do not just block the initiation of the extrinsic apoptotic pathway, but also result in increased survival after TCR engagement and protect from spontaneous apoptosis [66].

A dose-dependency of CD95 ligation had been observed earlier for CD95-sensitive SKW6.4 cells [65,67]. Lavrik and colleagues showed that a strong CD95 stimulation initiated death of this EBV-transformed lymphoblastoid B cell line, whereas CD95 stimulation below a certain threshold level triggered cFLIP-dependent survival associated with MAPK and NF-κB activation. In fact, these experimental observations on life and death decisions and also on the role of cFLIP isoforms in this context fit nicely into the computational models that have been developed in a systems biology approach to better understand CD95 signaling consequences [65,67-69].

**Figure 4:**
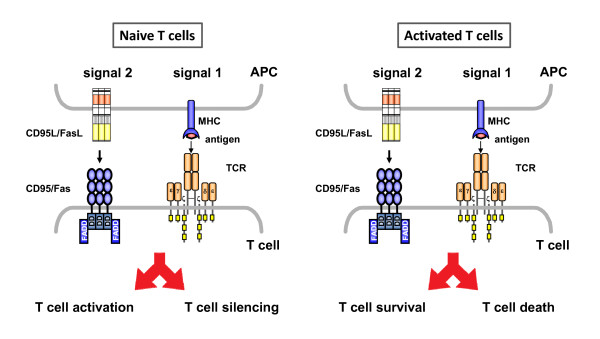
**Modulation of T cell responses through CD95 in naïve versus activated T cells**. The activation state of a given T cell (population) defines the signal threshold for pro- or non- apoptotic CD95 signaling. At the next level, the signal strength passing through CD95 determines whether signal transduction results in cell death, survival, cell cycle arrest or enhanced proliferation. In naïve CD95-resistant T cells, CD95 acts as a potent costimulatory receptor that can transduce activatory or inhibitory signals depending on the dose of CD95 agonists to modulate TCR/CD3 signal induction. Activated T cells are CD95-sensitive and undergo apoptosis when exposed to high concentrations of CD95L. In contrast, a weak CD95 stimulus (again below a certain threshold level) might induce survival signaling in the absence of detectable cell death.

Based on our results in primary T cells, we proposed a comparable threshold mechanism in primary human T cells [39]. However, since freshly isolated T cells display apoptosis resistance even to high concentrations of soluble or immobilized CD95 agonists (in contrast to SKW6.4 cells or activated T cells), CD95 signaling shifts towards inhibition of cell proliferation as initially emphasized by Strauss et al. [59] or Chen et al. [58]. As a consequence, high concentrations block TCR signal initiation (without inducing cell death), whereas low concentrations result in a sustained amplification of the TCR-induced activation processes mentioned above (Figure 4).

Notably, differential CD95 ligation might also determine cell fate and apoptosis sensitivity outside the immune system. Schüngel and colleagues recently reported that depending on the efficacy of CD95 receptor activation or the strength of the CD95L signal (e.g. using monoclonal anti-CD95 antibodies or hexameric CD95L, respectively), hepatocytes and non-parenchymal liver cells can either behave as type I cells (following strong CD95 receptor activation) or as type II cells where the BH3-only protein Bid amplifies the weak death receptor signal to render the liver cells more sensitive to CD95-induced apoptosis [70].

### What could be the physiological relevance of dose-dependent CD95 signaling in T cells?

The dose-dependency of CD95 signals calls for a closer look at the regulation of CD95L expression in cells that come into contact with resting and/or activated CD95 expressing T cells. Still, the role of CD95L expressed on epithelial cells of the thymus is controversially discussed. Some studies suggested a role of CD95L and/or CD95 during positive or negative selection [71-73]. However, most studies are again based on the *gld- *and *lpr-*models and describe phenomenology without providing detailed information about signaling alterations and consequences.

Although CD95L has been initially described as an inducible, more or less T cell specific, molecule, several studies document that also outside the thymus, CD95L is expressed on B cells [74], on APC including macrophages, 'dendritic cells' (DC) and Langerhans cells [75-77] and on tumour cells of different origin. With regard to tumour cells, (high level) CD95L expression might not only protect the tumour by inducing apoptosis in activated ("CD95-sensitive") tumour infiltrating cells, a mechanism that was referred to as 'tumor counterattack' [78], but could also block the activation of potentially tumour-reactive primary T cells for instance during metastatic spreading.

Regarding B cells or APC, it has been suggested that on those cells, CD95L might be upregulated in the course of an immune response or during cell maturation by yet unknown mechanisms accompanying antigen processing and presentation [75,79,80]. Thus, APC initially express low levels of CD95L. At the onset of an adaptive immune response to foreign antigens, the antigen presentation to naive T cell may therefore be associated with a costimulatory CD95 signal and result in enhanced activation and proliferation, as seen *in vitro *with suboptimal TCR stimulation in the presence of low amounts of CD95 agonists [39]. In this situation, CD95 triggering would support T cell expansion and the generation of effector T lymphocytes, which at the same time produce more 'T helper1' (Th1-)-type cytokines for the activation of 'natural killer' (NK) cells, macrophages and 'cytotoxic T cells' (CTLs). As a result of pathogen elimination, the expansion phase is followed by a contraction phase in which T cell numbers decline and reach their normal basal level. The reduction in T cell numbers could be accomplished and stabilized by the induction of apoptosis (AICD) in activated T cells on one hand and by preventing further activation of naive T cells on the other hand. According to the report by Strauss and colleagues [59] and our own observations using high doses of the CD95 agonists, this blockade could be achieved by high levels of CD95L, potentially on any neighboring cell. The recent reports therefore suggest that under physiological conditions, both the expression levels of CD95L and the threshold levels for CD95 "signal conversion" might be more relevant for the regulation and fine-tuning of the immune response than hitherto anticipated.

Along this line, it has been demonstrated that macrophages upregulate CD95L during an ongoing HIV-infection [81,82] and thereby contribute to the apoptotic depletion of uninfected CD4^+ ^T cells [83,84]. However, according to the report by Strauss et al. [59], another mechanism of reducing T cell numbers during HIV infection might be the block of proliferation of resting cells. Of course, the outcome of CD95 ligation very much depends on the state of activation of the respective T cell population. Infection with *Cryptococcus neoformans *results in an upregulation of CD95L on 'glucuronoxylomannan'/'toll-like receptor-4' (GXM/TLR-4)-triggered macrophages, associated with increased apoptotic T cell death in activated cells. As expected, also under such conditions, cell death induction is drastically reduced employing naïve T cells [85]. Likewise, CD95L-expressing DC trigger apoptosis of pre-activated cells but induce resistance of naïve CD4^+ ^and CD8^+ ^T cells [86]. Under pathophysiological conditions, increased levels of CD95L might prevent initial T cell expansion. Respective scenarios have not only been reported for HIV [59], but also for 'cytomegalovirus' (CMV) [87], measles virus [88] and 'herpes simplex virus' (HSV) [89]. Raftery and colleagues [87] observed that CMV-infected DC not only down-modulate MHC molecules but also upregulate CD95L, thereby inducing cell death of activated T cells and non-deletional suppression of the surviving T cells. Thus, also an upregulation of CD95L on infected cells may provide an effective immune escape mechanism for certain pathogens. Recently, Puliaeva and colleagues further suggested that the CD95/CD95L-system also plays a role in T cell/T cell-interactions. They showed in an *in vivo *mouse model that CD95 expression on CD4^+ ^T cells provides an important signal for CD4^+ ^T cell expansion and is required for optimal function of CD8^+ ^effector CTL [90].

## Conclusion

CD95 belongs to the TNFR superfamily and is best known for its capacity to execute cell death in CD95-sensitive cells. In this context, CD95-induced apoptosis plays an essential role in the maintenance of immune homeostasis and tolerance and in immune response termination. Dysregulation of pro-apoptotic functions contributes to several diseases including cancer or autoimmune syndromes and immunodeficiencies. In addition, non-apoptotic functions of CD95 in different cell types regulate proliferation, differentiation or chemotaxis. Also in T lymphocytes, CD95 acts as a dual-function receptor that conveys its differential signals depending on the cellular microenvironment and the state of activation. From the most recent data, we conclude that low levels of CD95L on APC positively costimulate naïve T cells and thus support the expansion phase. In contrast, when CD95L is upregulated during the immune response, this may eventually result in the induction of apoptosis in activated cells and the prevention of activation of resting cells. Under pathophysiological conditions, the threshold levels might be shifted to higher expression of CD95L as a mechanism of immune evasion of certain pathogens.

## Abbreviations

AICD: activation-induced cell death; APC: antigen-presenting cell; Bcl-X_L_: B-cell lymphoma-extra large; CD95L: CD95 ligand; cFLIP: cellular FLICE (caspase 8)-like inhibitory protein; cFLIP_S/R_: cFLIP_short/Raji_; cFLIP_L_: cFLIP_long_; CTL: cytotoxic T cell; DC: dendritic cell; DISC: death-inducing signaling complex; IFNγ: interferon γ; IκB: inhibitor of NF-κB; IL-2: interleukin-2; MAPK: mitogen activated protein kinase; NF-κB: nuclear factor 'kappa-light-chain-enhancer' of activated B-cells; PARP: poly (ADP-ribose) polymerase; PLCγ: phospholipase Cγ; TCR: T cell receptor; TNF: tumor necrosis factor; TNFR: TNF receptor; TRAF: TNF receptor-associated factor.

## Competing interests

The authors declare that they have no competing interests.

## Authors' contributions

MP designed and wrote the initial version of the article based on data obtained during the experimental work for completion of her doctoral thesis. The work was initiated and supervised by OJ. MP and OJ drafted the manuscript. Both authors read and approved the manuscript.
